# *Escherichia coli* Strains Display Varying Susceptibility to Grazing by the Soil Amoeba *Dictyostelium discoideum*

**DOI:** 10.3390/microorganisms11061457

**Published:** 2023-05-31

**Authors:** Gitanjali NandaKafle, Lane A. Blasius, Tarren Seale, Volker S. Brözel

**Affiliations:** 1 Department of Biology and Microbiology, South Dakota State University, Brookings, SD 57006, USA; gitanjalinandakafle2013@gmail.com (G.N.); lane.blasius@jacks.sdstate.edu (L.A.B.); 2 Department of Biochemistry, Genetics and Microbiology, University of Pretoria, Pretoria 0004, South Africa; tarren.seale@fabi.up.ac.za

**Keywords:** soil, *Escherichia coli*, grazing, amoeba, *Dictyostelium*, genome

## Abstract

Recent studies have shown that *Escherichia coli* can survive in different environments, including soils, and they can maintain populations in sterile soil for a long period of time. This indicates that growth-supporting nutrients are available; however, when grown in non-sterile soils, populations decline, suggesting that other biological factors play a role in controlling *E. coli* populations in soil. Free-living protozoa can affect the bacterial population by grazing. We hypothesized that *E. coli* strains capable of surviving in non-sterile soil possess mechanisms to protect themselves from amoeba predation. We determined the grazing rate of *E. coli* pasture isolates by using *Dictyostelium discoideum*. Bacterial suspensions applied to lactose agar as lines were allowed to grow for 24 h, when 4 μL of *D. discoideum* culture was inoculated in the center of each bacterial line. Grazing distances were measured after 4 days. The genomes of five grazing-susceptible and five grazing-resistant isolates were sequenced and compared. Grazing distance varied among isolates, which indicated that some *E. coli* are more susceptible to grazing by protozoa than others. When presented with a choice between grazing-susceptible and grazing-resistant isolates, *D. discoideum* grazed only on the susceptible strain. Grazing susceptibility phenotype did not align with the phylogroup, with both B1 and E strains found in both grazing groups. They also did not align by core genome phylogeny. Whole genome comparisons revealed that the five most highly grazed strains had 389 shared genes not found in the five least grazed strains. Conversely, the five least grazed strains shared 130 unique genes. The results indicate that long-term persistence of *E. coli* in soil is due at least in part to resistance to grazing by soil amoeba.

## 1. Introduction

*Escherichia coli* is well known as a species of diverse pathogenic and benign strains associated with the gastrointestinal tract and other mammalian environments [1,2]. It is widely used as indicator of fecal pollution of water, sediments, and soils [3]. In addition to the mammalian gastrointestinal tract, it can be isolated from an array of environments including soil, freshwater sediments, water plants, and even beach sand [4,5,6]. While *E. coli* are thought to decline once introduced into the extra-host environment, some *E. coli* have adapted to a lifestyle outside of the gastrointestinal tract, such as capsulated strains found in reservoirs [7], or a population in alpine grassland soil [8]. This occurs even when there is no evidence for re-introduction. There is evidence for selection of *E. coli* after introduction to soil, for example, in cattle pastures, where some strains introduced from bovine feces establish populations, whereas others appear to decline [9]. Population maintenance of *E. coli* in soils appears to be influenced by soil conditions such as soil chemistry [10], but the factors underlying strain-specific population maintenance are not yet well understood. 

Population maintenance of *E. coli* in an open environment is impacted by multiple factors, including the ability to grow within the niche, longevity or long-term stationary phase, resistance to stresses, fitness under competition with autochthonous microorganisms, and susceptibility to predation. We have demonstrated that a wide array of *E. coli* and other bacteria are able to grow using water-soluble nutrients available in soil [11,12]. Recently, we reported that diverse *E. coli* grown in liquid soil extract displayed long-term survival in the stationary phase (NandaKafle et al., submitted). In contrast to the GASP phenotype [13], these populations did not display a decline phase, with the entire population surviving in a culturable state. *E. coli* populations decline more in natural soils than in sterilized soils devoid of competitors [14,15]. They have been found to be susceptible to competition by autochthonous bacteria [16], but in other studies competition was reported to play a lesser role [16,17]. *E. coli* decay was minimal in outdoor microcosms that were exposed to natural UV radiation when the natural microbiota (predation and competition) was removed by disinfection [18]. This suggests that the natural microbiota play an important role in controlling *E. coli* population density in the environment. Taken together, some members of the species appear well attuned to maintain populations in soils in the face of challenges such as stress, competition, and predation. 

The global terrestrial biomass is estimated to comprise 7 Gt carbon of bacteria, 0.5 of archaea, 12 of fungi and 1.6 of protists [19]. Bacterial ecology has focused much on interactions among bacteria, and to a lesser extent with fungi, but the role of protozoa in bacterial population dynamics is not well understood. Predation contributes to decreases in bacterial population density [20]. Predation requires three consecutive stages; recognition or sensing, internationalization or ingestion, and digestion [21]. Amoeba have evolved mechanisms to find, ingest, and digest bacteria [22]. Conversely, bacteria have evolved strategies to evade or resist predation by protozoa [23]. *E. coli* are reportedly susceptible to predation by protozoa [24], but some strains adapt phenotypically to resist predation [25]. *E. coli* has been found to be an excellent food source for *Acanthamoeba polyphaga*, *A. castellanii*, and *H. vermiformis* [26], and for *Dictyostelium discoideum* [17,24]. Various protozoa isolated from dairy wastewater have been reported to have different grazing effects on *E. coli* [27]. The bacterial elimination rate by natural protozoa varies depending on different bacterial characteristics such as cell size, cell wall composition, presence of virulence factors, and location [28,29,30,31]. There are various defense mechanisms that bacteria can use to either avoid or endure predation [23]. Surface properties contribute to grazing susceptibility. Curli-negative *E. coli* O157 were able to survive predation more than curli-decorated variants [32]. Some serotypes of *Salmonella enterica* are more resistant to predation by amoeba than others [33,34]. The swarming motility of *Pantoea ananatis* BRT175, as impacted by *rhlA* and *rhlB,* involved in glycolipid surfactant biosynthesis, impacts it susceptibility to grazing [35]. Virulence factors of *E. coli* have also been shown to provide protection against predation from bactivorous protozoa [31]. *E. coli* O157:H7 and ExPec, carrying virulence genes *iroN, irp2,* and *fyuA,* involved in iron uptake were more resistant to grazing by *D. discoideum* than commensal *E. coli* [28,36]. Some reports have, however, indicated that both commensal and fecal indicator *E. coli* are equally susceptible to predation [37,38]. Whereas *stx*-encoding prophages of *E.coli* O157:H7 were reported to provide protection against predation by grazing protozoa [31], neither Stx nor the products of other bacteriophage genes affected predation by *Paramecium caudatum* or *Tetrahymena pyriformis* when using *E. coli* O157:H7 (EDL933D) and its isogenic mutant [39]. Protozoan predation is thus an important factor in shaping the genotypic and phenotypic structure of planktonic and terrestrial bacterial communities [40,41]. 

We have previously reported that some *E. coli* introduced into pasture soil maintain populations, whereas others appear to decline [9]. Isolates from maintained and declining populations were able to grow in liquid pasture soil extract, and all displayed long-term survival in the stationary phase with no detectable decline phase (NandaKafle et al., unpublished). We hypothesized that *E. coli* strains that maintain populations in pasture soil are less susceptible to amoeboid grazing. Therefore, we determined the grazing susceptibility of 363 *E. coli* isolates obtained in a pasture study to *D. discoideum*. We find that *E. coli* vary widely in susceptibility to grazing.

## 2. Materials and Methods

### 2.1. Strains Evaluated

*E. coli* were previously isolated from an enclosed 12.14 ha pasture at Volga, SD, USA (GPS co-ordinates 44°22′17.70″ N 96°58′1.54″ W). The pasture hosted cattle for one month during July every year [9]. Samples were collected from soil cores taken during June 2013 before cattle were introduced (soil before grazing, SBG, 45 isolates), soil cores during grazing (120 isolates), run-off during grazing (163 isolates), and from fresh cattle feces (35 isolates). Isolates were stored at −80 °C in 50% glycerol. *E. coli* MG1655 (K12), 933D (O157:H7), and TW 10509 (clade I) were included as controls. The amoeba *D. discoideum* were obtained from Carolina Biological Supply.

### 2.2. Culturing Conditions

*E. coli* isolates were recovered from −80 °C glycerol stocks on LB agar overnight and then inoculated into modified HL5 medium, then incubated at 28 °C while shaking (180 rpm) overnight [28]. Modified HL5 medium contained 10 g L^−1^ protease peptone (in place of Thiotone E, discontinued), 10 g L^−1^ glucose, 5 g L^−1^ yeast extract, 0.35 g L^−1^ Na_2_HPO_4_ 7H_2_O, 0.35 g L^−1^ KH_2_PO_4_, pH 6.5. Cells were washed once and re-suspended in HL5 medium, and the optical density was adjusted to A_546_ 0.50. Amoeba were cultured in 50 mL modified HL5 medium at 24 °C in a shaking incubator overnight, and cells were washed once. Initially modified HL5 agar medium was used to pre-culture *D. discoideum*, but we could not detect a grazing effect, and no fruiting bodies were formed. Various alternative culture media were evaluated, including LB, R2A, and LA (lactose agar). *D. discoideum* cells developed fruiting bodies on LA medium (1 g L^−1^ lactose, 1 g L^−1^ proteose peptone, and 20 g L^−1^ agar), a condition when there is no availability of immediate food source for the amoeba. 

### 2.3. Grazing Assay

**Figure 1:**
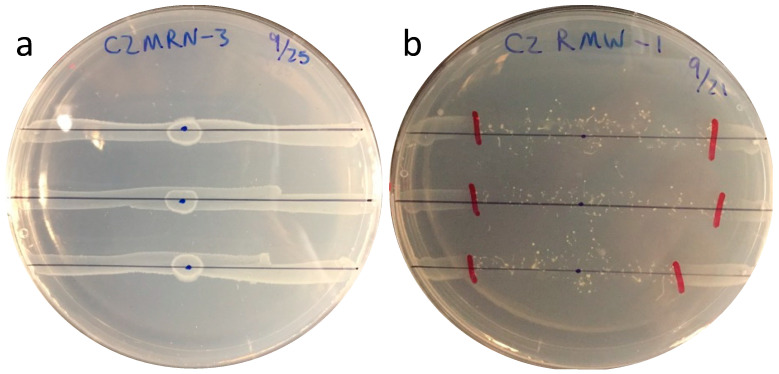
Growth of *E. coli* lines on LA agar after 24 h at 25 °C, and with *D. discoideum* applied at center (**a**), and after a further 96 h incubation at 25 °C in the dark (**b**).

All *E. coli* isolates (363) were evaluated for their susceptibility to grazing by using a quantitative assay as described by [42] with modifications. For this assay, *D. discoideum* was co-cultured with *E. coli* on LA plates. To test a particular isolate, 4 μL (A_546_ 0.50) bacterial suspension was applied to plates as three parallel lines spread across the plate (Figure 1a), and incubated for 24 h at 22 °C. Four microliters of *D. discoideum* broth culture was inoculated at the center of each line (Figure 1a). All plates were incubated at 22 °C for 4 days in the dark. The proliferating (grazing) fronts advanced along the bacterial lines (Figure 1b). The distance of amoeba grazing was measured in millimeters. To determine the difference in grazing susceptibility among the four sample types, an ANOVA test was performed using the R program [43].

### 2.4. Grazing Preferences by Amoeba

For grazing preference determination, we chose two highly grazed (greatest grazing distance) and six least grazed strains (smallest grazing distance). Each susceptible strain was inoculated on an LA plate with a resistant strain. Four μL of culture of each was streaked on the plate as diverging straight lines touching each other at one end to create a V shape. After 24 h incubation, 4 μL of amoeba suspension was then placed at the base of the diverging bacterial lines. Plates were incubated at room temperature for 4 days and grazing distances were measured.

### 2.5. Genome Analysis of Most and Least Grazed Isolates

Genomic DNA was extracted from overnight LB agar cultures suspended in 10 mM phosphate buffer (pH 7.0) using the genomic DNA Quick Prep Kit (Zymo Research, Irvine, CA, USA), and all extracted DNA samples were quantified using a Nanodrop Spectrophotometer (ThermoFisher, Waltham, MA, USA) as well as a Qubit Fluorometer (ThermoFisher, Waltahma, MA, USA). The DNA samples were sent to Microbes NG, UK for sequencing (http://www.microbesng.com, accessed April 2016), which is supported by the BBSRC (grant number BB/L024209/1). The protocol used for sequencing is briefly explained; the Genomic DNA libraries were prepared using Nextera XT Library Prep Kit (Illumina, San Diego, CA, USA) following the manufacturer’s protocol with the following modifications: two nanograms of DNA instead of one were used as input, and PCR elongation time was increased to 1 min from 30 s. DNA quantification and library preparation were carried out on a Hamilton Microlab STAR automated liquid handling system. Pooled libraries were quantified using the Kapa Biosystems Library Quantification Kit for Illumina on a Roche light cycler 96 qPCR machine. Libraries were sequenced on the Illumina HiSeq using a 250 bp paired end protocol. Reads were adapter-trimmed using a Trimmomatic 0.30 with a sliding window quality cutoff of Q15 [44]. De novo assembly was performed on samples using SPAdes version 3.7 [45], and contigs were annotated using Prokka 1.11 [46]. 

Annotated genomes were uploaded to the EDGAR 3.0 platform for comparative genome analysis [47]. The core genome was determined on EDGAR 3.0 using 23 *Escherichia* genomes [47]. Two genomes representing clade I, four *E. ruysiae*, one *E. marmotae*, three *E. albertii* and one *E. fergusonii* genomes were used as an outgroup. The core genes were aligned using the MUSCLE plugin from the CLC Main Workbench 7.0 [48] (www.qiagenbioinformatics.com, accessed on 26 April 2023). The core genes were concatenated and partitioned using FASconCAT-G 1.02 and ProtTest 3.4, respectively [49,50]. ProtTest 3.4 was used to determine a model for each gene separately. A core maximum likelihood phylogenetic tree of the core genomes was drawn using RAxML with 100 bootstrap replicates [51]. The pangenome was determined using EDGAR 3.0. The core genes were removed to yield the accessory or non-core genes, and a UPGMA dendrogram constructed using PAST 3 (Paleontological Statistics Software Package for Education and Data Analysis) with the Jaccard similarity index [52]. 

### 2.6. Genes Associated with Grazing Susceptibility of E. coli Isolates

To identify factors contributing to grazing susceptibility, the genomes of five of the least grazed and five of the most grazed isolates were sequenced. These were designated least grazed group (LGG) and highly grazed group (HGG). Genes common to either LGG or HGG or common to both groups were identified using the EDGAR bioinformatics platform [53] and R program [43]. 

## 3. Results

### 3.1. Grazing Susceptibility

Grazing susceptibility of *E. coli* isolates from soil, run-off, soil before grazing (SBG), and bovine feces was determined by the grazing distances of *D. discoideum* introduced at the center of *E. coli* culture lines on lactose agar (Figure 1). Grazing distance after 96 h varied widely among the various *E. coli*; between 0 and 7 cm from the point of inoculation. This indicated that susceptibility of *E. coli* to grazing by *D. discoideum* was strain specific. 

**Figure 2:**
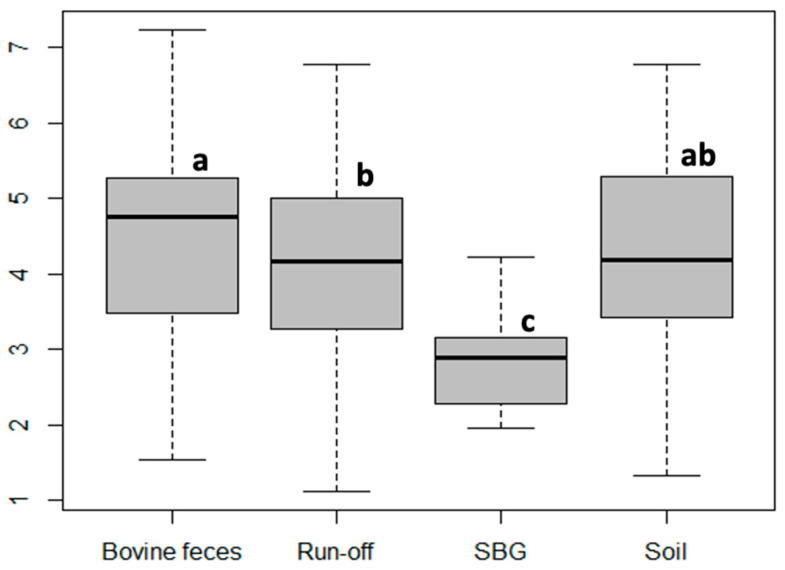
Box and whisker plot depicting the grazing distances of *D. discoideum* on *E. coli* isolates from different environmental sources. Sample groups with the same letter were not significantly different as determined by ANOVA.

The distribution of grazing susceptibility varied significantly among the four sample sources (Figure 2). The SBG isolate group showed the lowest susceptibility to grazing by *D. discoideum*. The SBG isolates represented strains that were able to persist in soil over a full year [9]. The lower susceptibility to grazing of SBG isolates compared to soil, run-off, and feces isolates suggests that population maintenance in soil was due, at least in part, to persistence in the presence of grazing protozoa. 

### 3.2. Grazing Preferences by D. discoideum

**Figure 3:**
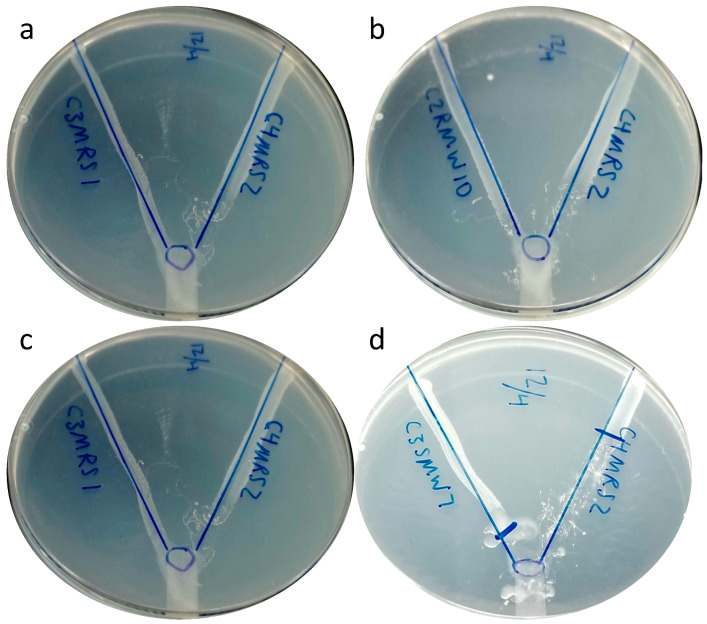
Grazing preference of *D. discoideum* between grazing-susceptible (right side) and grazing-resistant strains (left side) of *E. coli*. Examples shown include the isolate pairs C3MRS1 and C4MRS2 (**a**), C2RMW10 and C4MRS2 (**b**), C3MRS1 and C4MRS2 (**c**) and C3SMW7 and C4MRS2 (**d**).

When *D. discoideum* was grown in the presence of two *E. coli* isolates of different grazing susceptibility (LGG or HGG), it preferred HGG over LGG strains. The grazing was initiated first on the highly susceptible isolates, where it grazed a longer distance; later, it grazed on the least susceptible isolates (Figure 3). Our results indicate that *D. discoideum* grazes on both isolates, but displays a preference for strains that are highly susceptible to grazing.

### 3.3. Presence of Virulence Genes and Grazing Susceptibility 

To determine if there is any relationship between the presence of virulence genes and the grazing susceptibility of *E. coli*, the presence of six virulence genes was correlated with grazing distance. We had previously determined the presence of *stx1, stx2, eaeA*, *hlyA*, ST, and LT in each isolate by PCR (data not shown). There was no significant correlation found between grazing susceptibility and virulence gene prevalence (R^2^ = 0.1597) (Supplementary Figure S1).

### 3.4. Genomes of Grazing-Susceptible versus -Resistant Isolates

**Figure 4:**
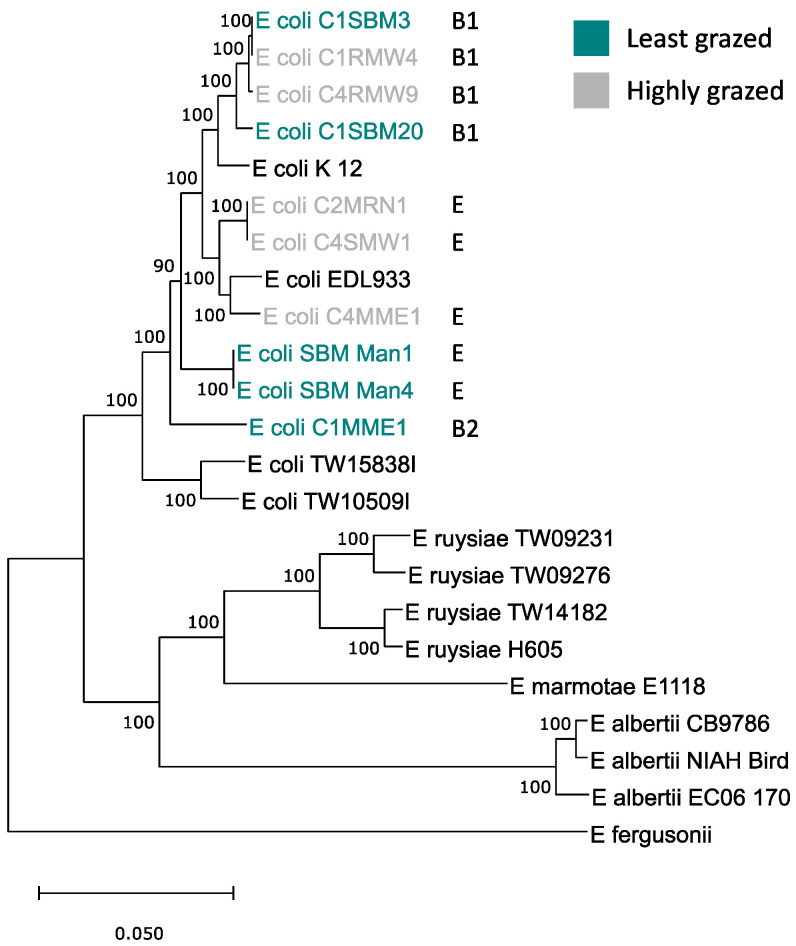
Maximum likelihood phylogenetic tree of the core genomes of five highly and least grazed *E. coli* isolates compared to other strains of *E. coli* and other species of the genus. Letters identify the respective phylogroups of isolates.

**Figure 5:**
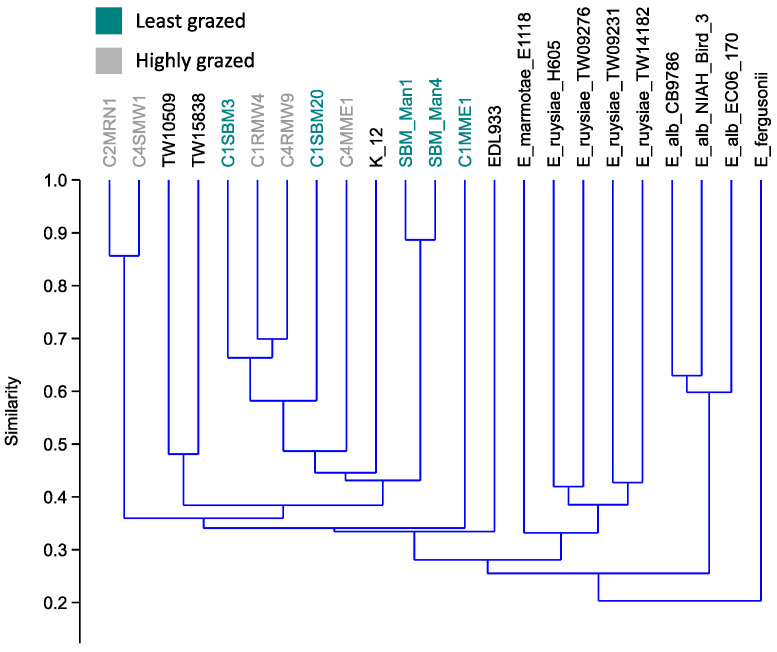
UPGMA dendrogram of accessory or non-core genes of the highly and least grazed *E. coli* isolates, compared to other strains of *E. coli* and other species of the genus.

Genome comparisons were conducted to determine whether predation resistance in the LGG might be due to differences in genotype or phylogeny. The five highly and least grazed isolates were all shown to be true *E. coli* by core genome phylogeny (Figure 4). The average genome size for the least grazed group (LGG) was 4852 genes and the highly grazed group (HGG) had 5100 genes. Yet, the grazing susceptibility phenotype did not align with phylogeny or phylogroup (Figure 4). The five highly and least grazed isolate groups both had members of phylogroups B1 and E. Likewise, highly and least grazed isolates did not separate by non-core or accessory genome content, with isolates occurring among each other on the tree (Figure 5). Collectively, genome content overall did not align with grazing susceptibility, suggesting that grazing susceptibility is not based on the phylogeny of isolates. 

**Figure 6:**
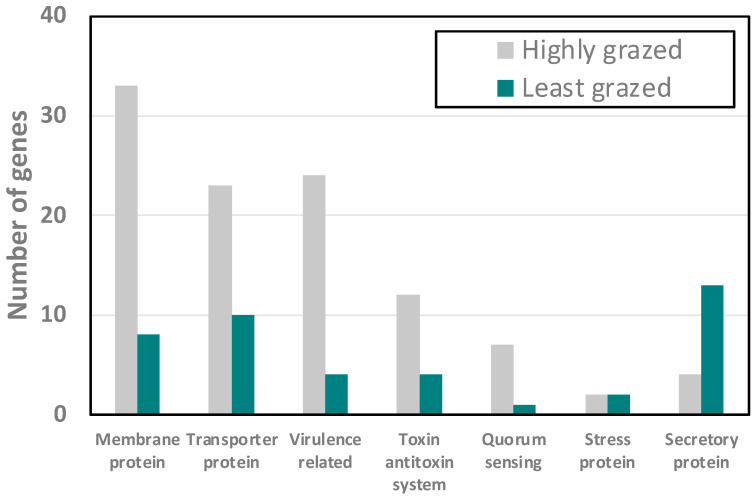
Number of genes uniquely common to the five highly grazed versus five least grazed *E. coli* associated with grazing susceptibility.

To determine whether there were genes common to either group of isolates, we looked for uniquely shared genes. The highly grazed group had 389 genes specific to their group, more than double the 130 unique genes shared by the LGG. These unique genes were grouped based on their function (Figure 6) and are listed in Table 1.

The number of unique-membrane-related genes in the highly grazed group was 33 and in the LGG it was only 8, suggesting a substantial difference in their membrane composition. These included multiple outer membrane proteins which could act as specific surface molecules for recognition. The HGG contained 23 transporter genes compared to the LGG, with only 10. Surprisingly, we found that there were only 4 secretory-protein-related genes in the HGG, and they were related to the type III secretory system, whereas in the LGG there were 10 secretory system type-II-protein-related genes present. This points to differences in effectors excreted directly into potential eukaryotic cells (type III) by the LGG, and effectors secreted outside the cell in the HGG. The HGG also possessed many fimbial and flagellar genes, and small toxic proteins and hemolysin genes. The LGG had only three fimbrial and invasion-related genes, suggesting that the HGG could possibly contribute more virulence genes compared to the LGG. There were also a high number of toxin–antitoxin system genes in the HGG compared to the LGG. Quorum sensing molecules such as autoinducer-2-related genes were more abundant in the HGG compared to the LGG, where only one autoinducer 2-binding protein gene, lsrB, was present. Our results suggested that the LGG and HGG strains are phenotypically and genotypically different from each other in surface properties, proteins excreted, and signaling molecules. 

## 4. Discussion

*E. coli* isolates from the cattle pasture showed different susceptibilities to protozoan predation. The majority of isolates from SBG samples, considered as environmental [9], showed significantly higher resistance to grazing compared to soil, run-off, and bovine feces isolates. This indicates that *E. coli* maintaining long-term populations in soil either lack traits that render the HGG susceptible or display traits warding off the grazing amoeba. Lesser susceptibility of the LGG to grazing was supported when *D. discoideum* was presented with a choice between pairs of LGG and HGG strains. The grazers consistently selected the HGG strain in each pair (Figure 3). Adiba et al. [28] have shown that *D. discoideum* was able to survive and phagocytize *E. coli* strains not harboring virulence genes involved in iron capture (*iroN, fyuA, irp*), not resistant to bile, serum, or lactoferrin, or that do not belong to phylogroup B2. 

In our study, interestingly, we also found that isolates belonging to the B2 phylogroup showed resistance to protozoan grazing, although there were very few B2 isolates in our collection (in total, 368 isolates and only 9 B2 isolates). The highest grazing distance was 7.2 cm and the range of grazing distance for isolates belonging to phylogroup B2 was 0–3.1 cm. It has been shown that *E.coli* strains that harbor virulence genes are able to survive and replicate in common environmental protozoa such as *E. coli* O157 [31,54], or extra-intestinal pathogenic *E. coli* [28]. To determine the correlation between the presence of virulence genes and grazing resistance, we detected the presence and absence of six virulence genes in all isolates. We did not find any correlation between the presence of virulence genes and grazing resistance of *E. coli.* We also measured the grazing distance of *E. coli* O157:H7 strains and did not find any significant resistance by the strain. Our result is consistent with Schmidt, Shringi, and Besser [39], who reported that *P. caudatum* consistently reduced both *E. coli* O157:H7 (EDL933D) and non-Shiga-toxin-cattle-commensal *E.coli* populations by 1–3 log CFU when grown together in broth culture over three days at an ambient laboratory temperature. 

If virulence genes are not the major factor for *E. coli* to be resistant to predation, then what are the traits responsible for their ability to evade grazing instead? To find out the difference between the least grazed isolates (resistant isolates) and the highly grazed group (susceptible isolates), we chose five isolates from the least grazed group (denoted as LGG) and five isolates from the highly grazed group (HGG) and sequenced these to compare their genome data. We found that the two groups shared a core genome consisting of 3414 genes, while each group also has some unique genes they do not share. The core genomes did not group into HGG versus LGG, indicating that grazing susceptibility was not due to variations among sequences of core genes, but rather due to the presence or absence of specific genes. The HGG had 389 shared genes not occurring in any of the LGG sequenced, and the LGG had 130 unique shared genes. It was interesting to see that the HGG has a higher abundance of membrane protein, transporter protein, fimbrial protein, flagellar protein, toxin–antitoxin-system-related protein, and autoinducer-2. However, the LGG has a high number of secretory-system-II proteins compared to the HGG, which has fewer secretory system III proteins. 

A recent study by Snyder, et al. [55] found that mutant strains of *E. coli* that are resistant to *D. discoideum* phagocytosis possess several genes related to flagella, oxidoreductase, and acid resistance. These genes may have the potential to develop a mechanism to resist *D. discoideum* predation, which contributes to the selection and maintenance of bacterial virulence factors against mammalian hosts. *Salmonella enterica* subsp. Typhimurium inhibits the *D. discoideum* starvation response through the type III secretion system, thereby preventing sporulation [56]. The type-III secretion system in the HGG may also play a role in secreting substrate that may allow the starvation response of *D. discoideum*. Type II secretion systems occur in both pathogenic and non-pathogenic *E. coli,* and the output T2S secretory proteins can be a diverse group of toxins, degradative enzymes, and other effector proteins. This system is clearly used by bacteria for environmental survival and virulence [57]. This report suggests that the T2S system may play an important role in the LGG ‘s ability to resist predation. We also found autoinducer-2 related genes in the HGG, which are part of quorum-sensing system that allows communication with many different bacterial species [58]. It has also been reported that functional quorum sensing is important for the interaction of *Vibrio cholera* and the amoeba *A. castellanii.* Upon being phagocytized by the amoeba, *V. cholera* can resist intracellular killing [59]. The presence of autoinducer 2 in HGG indicates that the cells interact with *D. discoideum* to phagocytose. It may be possible that the cells are not completely killed, but form a symbiotic association with amoeba of farmer clones that carry bacteria through their social stages or dispersal stages, and can be identified by the presence of bacteria in their sorus [60]. It will be interesting to investigate the presence of *E. coli* cells in the sorus of *D. discoideum* that has grazed on HGG isolates.

Our study did not yield any detailed information about the association of genes specific to *E. coli* survival from protozoan predation. The presence of genes unique to thee HGG and LGG may play a role in the grazing susceptibility or grazing resistance of bacteria. To determine the role of these genes of protozoan predation, more investigation is needed. Our results, of a characterization of amoeba grazing on distinct *E. coli* isolates and a correlation between the presence of virulence genes and grazing resistance, deviate from previous reports [28,36]. These inconsistencies could be attributed to differences in amoeba clones, plating methods, nutrient conditions, and the laboratory atmosphere. In our study, we found that the plating medium clearly affects the growth of amoeba clones on distinct *E. coli* populations.

Population dynamics of *E. coli* in the environment have been studied widely from the perspectives of nutrient requirements, stationary phase physiology and stress response, and competition with other bacteria. In contrast, the role of amoeba in affecting population numbers through grazing has been little studied. Our results indicate that grazing by amoeba has a substantial effect on population densities of diverse *E. coli* in soils and sediments.

## 5. Conclusions

In conclusion, our study clearly depicts that there is a difference in the grazing susceptibility of *E. coli* isolates. The environmental *E. coli* that survived in the pasture without the presence of grazing animals were also significantly more resistant to grazing by *D. discoideum*. The highly grazed group contained a much larger number of genes encoding surface-related functions, such as membrane proteins and exporters than did the least grazed group. The results indicate that the long-term persistence of *E. coli* in soil is due, at least in part, to resistance to grazing by soil amoeba.

## Figures and Tables

**Table 1:** Genes uniquely common to the five most grazed (highly grazed group) versus five least grazed (least grazed group) *E. coli* associated with grazing susceptibility.

Highly Grazed Group—HGG (Susceptible)	Least Grazed Group—LGG (Resistant)
Membrane protein: 33	Membrane protein: 8
D-methionine-binding lipoprotein MetQ precursor, 00706	LPS assembly protein LptD, 02333
Outer membrane protein IcsA autotransporter precursor, 01754	Periplasmic trehalase, 04132
Outer membrane usher protein FimD precursor, 00603	Inner membrane protein YijD, 04745
Putative outer membrane usher protein ElfC precursor, 02959	Cryptic outer membrane porin BglH, 02531
Threonine-rich inner membrane protein GfcA precursor, 03321	Inner membrane protein YmfA, 02786
Putative lipoprotein GfcB precursor, 03320	Inner membrane protein YcfZ, 02787
Bestrophin, RFP-TM, chloride channel, 04701	Outer membrane usher protein PapC, 03086
Outer membrane usher protein FimD precursor, 04713	Major curlin subunit, 02865
Outer membrane porin protein OmpD precursor, 05195	
Inner membrane protein YnbA, 05048	
Outer membrane protein G precursor, 03566	
Inner membrane ABC transporter permease protein YcjP, 03559	
Inner membrane ABC transporter permease protein YcjO, 03558	
Inner membrane protein YnjI, 00078	
Inner membrane metabolite transport protein YdjE, 00071	
Inner membrane metabolite transport protein YdjE, 00065	
Inner membrane protein YedR, 04633	
Putative inner membrane protein, 04857	
Lipoprotein YlpA precursor, 05409	
Type IV conjugative transfer system lipoprotein (TraV), 05429	
Outer membrane protein IcsA autotransporter precursor, 03405	
Inner membrane protein YmgF, 03404	
InvH outer membrane lipoprotein, 02742	
Lipoprotein PrgK precursor, 02737	
Phospholipase YtpA, 05051	
Putative penicillin-binding protein PbpX, 00929	
Inner membrane protein YiaV precursor, 01085	
Inner membrane protein YiaW, 01084	
Inner membrane protein YidI, 03190	
Putative outer membrane usher protein ElfC precursor, 01369	
Inner membrane protein YihN, 01208	
Energy-coupling factor transporter transmembrane protein EcfT, 02823	
Inner membrane protein YhaI, 02855	
Transporter protein: 23	Transporter protein: 10
Putative transporter YycB, 03297	Ribose import permease protein RbsC, 00866
4-hydroxybenzoate transporter PcaK, 01955	Ribose import ATP-binding protein RbsA, 00865
Inner membrane transporter YcaM, 02919	Putrescine transporter PotE, 03286
Polysialic acid transport protein KpsD precursor, 03317	Autoinducer 2 import system permease protein LsrD, 00867
Putative autotransporter precursor, 03444	Electron transport complex subunit RsxC, 01281
Proline/betaine transporter, 04676	Fe^2+^ transporter FeoB, 02631
Sugar efflux transporter, 04692	L-fucose-proton symporter, 01383
Multidrug transporter EmrE, 00208	C4-dicarboxylate TRAP transporter permease protein DctM, 01554
Putative D,D-dipeptide transport system permease protein DdpB, 05180	D-galactonate transporter, 02501
Putative D,D-dipeptide transport ATP-binding protein DdpD, 05182	Sialic acid TRAP transporter small permease protein SiaQ, 01553
Putative D,D-dipeptide transport ATP-binding protein DdpF, 05183	
L-carnitine/gamma-butyrobetaine antiporter, 00040	
Putative autotransporter precursor, 04851	
Putative autotransporter precursor, 03444	
Yop proteins translocation protein F, 02739	
Sugar efflux transporter C, 00997	
Putrescine importer PuuP, 03543	
L-carnitine/gamma-butyrobetaine antiporter, 00040	
Putative formate transporter 1, 00871	
Inner membrane ABC transporter permease protein YtfT, 03372	
Energy-coupling factor transporter ATP-binding protein EcfA1, 02824	
Energy-coupling factor transporter ATP-binding protein EcfA1, 02825	
High-affinity glucose transporter, 02206	
Virulence related: 24	Virulence related: 4
Type-1 fimbrial protein, A chain precursor, 00605	Putative fimbrial-like protein YadM, 04227
Chaperone protein FimC precursor, 00604	Invasin, 00840
Putative fimbrial-like protein ElfG precursor, 00602	Putative fimbrial-like protein YbgD, 03085
Type-1 fimbrial protein, A chain precursor, 00601	Putative fimbrial-like protein YadM, 04227
Fimbrial A protein precursor, 01601	
Virulence factors putative positive transcription regulator BvgA, 00600	
Putative fimbrial chaperone protein ElfD precursor, 02958	
Fimbrial subunit ElfA precursor, 02957	
Putative fimbrial-like protein ElfG precursor, 02960	
Putative fimbrial-like protein YcbU precursor, 02961	
Putative fimbrial-like protein YcbV precursor, 02962	
Putative fimbrial chaperone YcbF precursor, 02963	
Hemolysin E, chromosomal, 03417	
IDENTICAL PARALOGS Small toxic polypeptide LdrD Small toxic polypeptide LdrD, 04573 & 04574	
S-fimbrial protein subunit SfaG precursor, 04714	
S-fimbrial adhesin protein SfaS precursor, 04715	
S-fimbrial protein subunit SfaH, 04716	
Flagellin, 02361	
Small toxic protein TisB, 03185	
Flagellar biosynthesis protein FliR, 02744	
Flagellar biosynthesis protein FliQ, 02745	
Flagellar biosynthetic protein FliP precursor, 02746	
Putative fimbrial-like protein ElfG precursor, 01370	
Type-1 fimbrial protein, A chain precursor, 01367	
Toxin antitoxin system: 12	Toxin antitoxin system: 4
Antitoxin DinJ, 00418	Toxin YoeB, 02097
Antitoxin ParD1, 03953	Antitoxin YefM, 02096
Antitoxin HicB, 01856	Toxin HigB-2, 04560
Antitoxin ParD1, 03953	Antitoxin HigA-2, 04561
Antitoxin VapB, 01875	
Antitoxin HipB, 04711	
Toxin ParE1, 03952	
Antitoxin MazE, 02588	
Antitoxin PrlF, 04802	
Toxin YhaV, 04801	
Antitoxin HigA, 02876	
Antitoxin ChpS, 03367	
Quorum sensing: 7	Quorum sensing: 1
Autoinducer 2-binding protein LsrB precursor, 04705	Autoinducer 2-binding protein LsrB, 00864
Autoinducer 2 import system permease protein LsrD, 04706	
Autoinducer 2 import system permease protein LsrC, 04707	
Autoinducer 2 import ATP-binding protein LsrA, 04708	
Transcriptional regulator LsrR, 04709	
Autoinducer 2 kise LsrK, 04710	
Autoinducer 2-degrading protein LsrG, 04703,	
Stress protein: 2	Stress protein: 2
Stress-induced bacterial acidophilic repeat motif protein, 00310	General stress protein A, 02418
General stress protein 69, 00069	General stress protein A, 02415
Secretory protein: 4	Secretory protein: 13
Secreted effector protein pipB2, 04251	Putative type II secretion system protein D, 04794
Type III secretion system protein SpaO, 02748	Type II secretion system protein E, 03006
Type III secretion system protein PrgH-EprH (PrgH), 02740	Type II secretion system protein F, 03005
Type III secretion system protein SpaO, 02748	Putative type II secretion system protein G, 04791
	Type II secretion system protein M, 02998
	Type II secretion system protein L, 02999
	Putative type II secretion system protein K, 03000
	Type II secretion system protein J, 03001
	Type II secretion system protein I, 03002
	Type II secretion system protein H, 03003
	Type II secretion system protein G, 03004
	Type II secretion system protein D, 03007
	Type II secretion system protein C, 03008

## Data Availability

Data available at http://edgar3.computational.bio accessed on 26 April 2023.

## Supplementary Materials

The following supporting information can be downloaded at: https://www.mdpi.com/article/10.3390/microorganisms11061457/s1, Figure S1: Correlation between grazing distance and presence of six pathogenic genes *stx1, stx2, eaeA, hlyA*, ST and LT.
