# Supplementary material for: Escherichia coli Strains Display Varying Susceptibility to Grazing by the Soil Amoeba Dictyostelium discoideum
Source: Microorganisms. 2023 May 31;11(6):1457. doi: 10.3390/microorganisms11061457 (PMC10304320; doi:10.3390/microorganisms11061457)
Supplement: Supplementary file 1 [file microorganisms-11-01457-s001.zip › microorganisms-2402261-supplementary.pptx]

## Slide 1
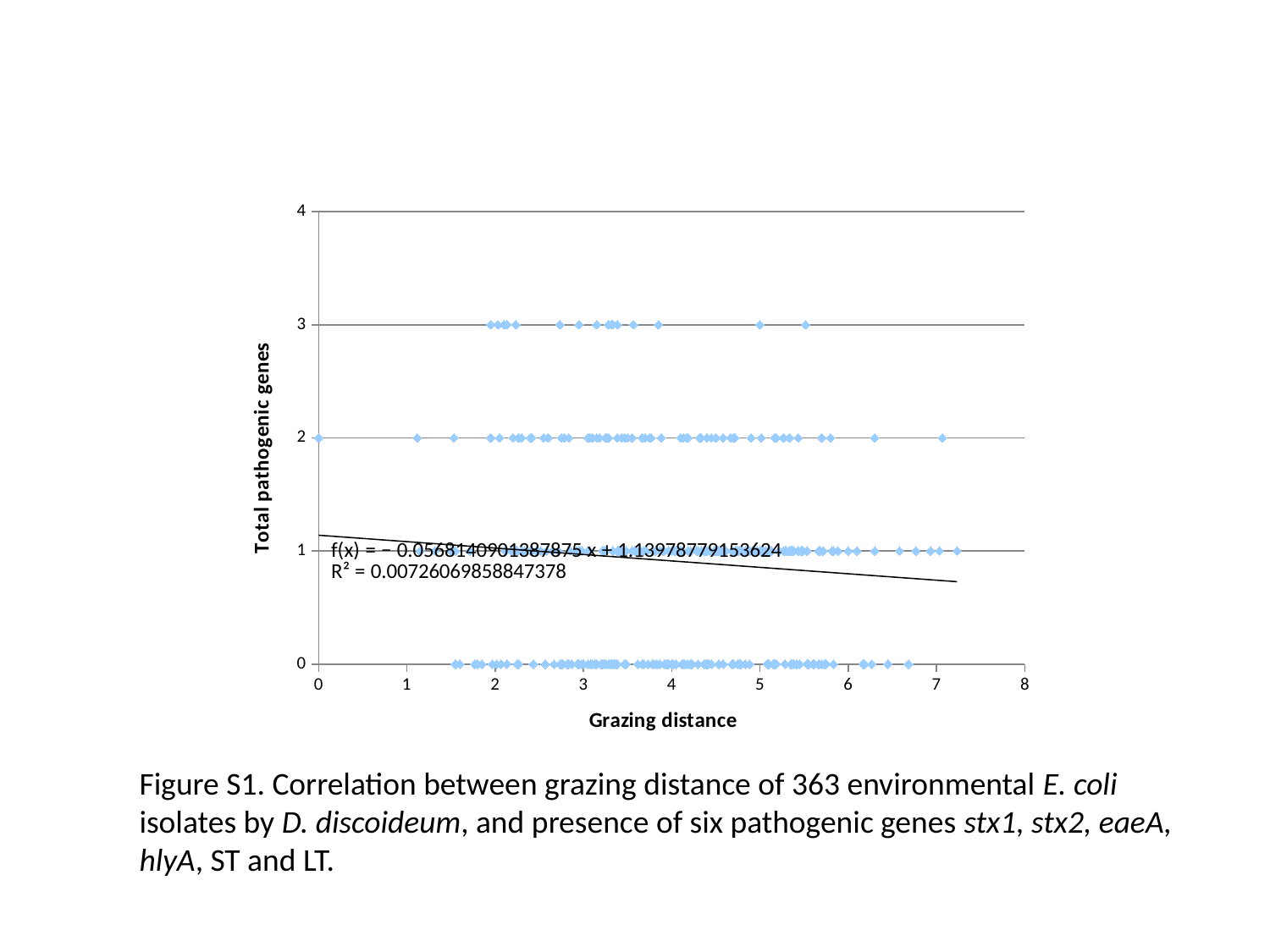

### Chart
| Category | |
|---|---|Figure S1. Correlation between grazing distance of 363 environmental E. coli isolates by D. discoideum, and presence of six pathogenic genes stx1, stx2, eaeA, hlyA, ST and LT.
